# Are We Ready to Get Rid of the Terms “Chalimus” and ”Preadult” in the Caligid (Crustacea: Copepoda: Caligidae) Life Cycle Nomenclature?

**DOI:** 10.3390/pathogens12030460

**Published:** 2023-03-14

**Authors:** Wojciech Piasecki, Balu Alagar Venmathi Maran, Susumu Ohtsuka

**Affiliations:** 1 Institute of Marine and Environmental Sciences, University of Szczecin, ul. Adama Mickiewicza 16, 70-383 Szczecin, Poland; 2 Borneo Marine Research Institute, Universiti Malaysia Sabah, Jalan UMS, Kota Kinabalu 88400, Malaysia; bavmaran@ums.edu.my; 3 Takehara Station, Setouchi Field Science Center, Graduate School of Integrated Sciences for Life, Hiroshima University, Takehara 725-0024, Japan; ohtsuka@hiroshima-u.ac.jp

**Keywords:** caligid, nauplius, copepodid, chalimus, preadult, ontogeny, life cycle, larva, juvenile

## Abstract

In view of recent studies, we suggest that the term “preadult” should not be used in scientific reports on Copepoda parasitic on fishes as having no explicit meaning or further justification. Consequently, the term “chalimus” with its use currently restricted in the Caligidae to at most two instars in the life cycles of species of *Lepeophtheirus*, also becomes redundant. In our new understanding, both the chalimus and preadult stages should be referred to as the respective copepodid stages (II through V, in integrative terminology). The terminology for the caligid copepod life cycle thereby becomes consistent with that for the homologous stages of other podoplean copepods. We see no justification for keeping “chalimus” and “preadult” even as purely practical terms. To justify this reinterpretation, we comprehensively summarize and reinterpret the patterns of instar succession reported in previous studies on the ontogeny of caligid copepods, with special attention to the frontal filament. Key concepts are illustrated in diagrams. We conclude that, using the new integrative terminology, copepods of the family Caligidae have the following stages in their life cycles: nauplius I, nauplius II (both free-living), copepodid I (infective), copepodid II (chalimus 1), copepodid III (chalimus 2), copepodid IV (chalimus 3/preadult 1), copepodid V (chalimus 4/preadult 2), and adult (parasitic). With this admittedly polemical paper, we hope to spark a discussion about this terminological problem.

## 1. Background of This Review

The class Copepoda, one of the most prominent taxa of the phylum Arthropoda, accommodates some 14,600 accepted species [1], and this number has been growing at a relatively fast pace. Copepods are ecologically very diverse and are almost equally represented by free-living as well as symbiotic species [2] (although this proportion may differ in most recent estimates). Many of the latter group, about 2400 valid species, are parasites of fishes [1]. Two factors indicate that this number is just the tip of the iceberg. The first is host specificity, which is usually narrow enough to allow individual fish species to have their own ”private” parasite species, some of which are copepods. Less specific parasites can be found on hosts representing the same genus or a family. By the end of 2022, FishBase reported 34,900 valid fish species [3], only a small percentage of which have been examined by parasitologists. Unfortunately, the majority of those researchers have focused on internal parasites (helminths) whereas externally occurring copepods, if present, have usually been overlooked or misidentified. Consequently, thousands of species of parasitic copepods potentially remain to be discovered and described. Until recently, progress in studies of those copepods was slow because of the limited practical effects of parasitic copepods on the economy. This situation changed with the onset of mariculture operations between the 1970s and 1990s. Formerly low-intensity infections of copepods occurring on individual fish, sparsely distributed in the ocean, received new horizons of opportunity. Crowding of fish in marine net pens provided excellent conditions for parasite transmission. The first copepods to take advantage of this situation were *Lepeophtheirus salmonis* (Krøyer, 1837) and *Caligus elongatus* von Nordmann, 1832 which began to excessively infect cultured Atlantic salmon, *Salmo salar* Linnaeus, 1758. When introduced to non-native areas (e.g., Chile), the latter fish species attracted local parasites that had earlier been unknown to science (e.g., *Caligus rogercresseyi* Boxshall et Bravo, 2000). Consumers’ demand to diversify seafood products in the marketplace has broadened the range of cultured fish species, thereby lengthening the list of problematic parasites, especially externally attached copepods collectively known as sea lice that belong to the family Caligidae. Currently, 517 valid species represent the family Caligidae [1]. It should be emphasized that the caligids differ substantially from other families of the Siphonostomatoida in the extent of their sexual dimorphism. Other siphonostomatoids tend to have dwarf males, of rather limited pathological effect on the host. Both sexes are usually permanently or semi-permanently attached to the fish. Conversely, adult males and females of many caligid copepods are relatively large and free-swimming. They are capable of changing host individuals and the location on a fish. This, in turn, increases their pathogenic potential. Differences between the sexes become visible at the stage of chalimus 3 or even earlier. Massive sea lice infections, resulting in serious economic losses, were initially combatted using organophosphate compounds, but when these proved to be environmentally harmful, political decision-makers allocated substantial funds to basic research on Copepoda in the hope of developing alternative control measures. This represented a serious boost for copepod science, especially benefitting researchers who were studying sea lice biology.

For centuries, the free-living copepods and the symbiotic ones were studied in isolation, utilizing different methodologies and terminology. Notable examples of publications that have recently contributed to the standardization of copepod terminology include those by Kabata [4], Dudley [5], Huys and Boxshall [6], and Boxshall and Halsey [7], which all dealt with many aspects of copepodology but mainly focused on morphology, taxonomy, and phylogeny. We believe the time has come to integrate also the nomenclatural systems that have been applied to the ontogenetic studies of Copepoda, a goal we will pursue herein.

We were motivated to write this polemical article by a statement of Z. Kabata, one of the doyens of copepodology who wrote, “The unceasing progress of science moves in an uncoordinated manner, more often than not following the lines of least resistance, or exploiting opportunities provided by technological breakthroughs. Stimuli are indiscriminately provided to various fields of science by new concepts or theories. Rapid progress on narrow fronts leaves behind lacunae of ignorance. It is advisable, therefore, to pause occasionally and to take stock, to marshal our achievements, assess our failures, and—hopefully—plot rational paths ahead.” (p. 2, [8]).

In this paper, we review all available literature on the life cycles of caligid copepods parasitic on fishes. We carefully analyze the descriptions and the terminology used. Our main goal is to understand the role and significance of some terms in relation to the most recent discoveries. Our intention is to reconcile the terminology used in ontogenetic studies on both free-living and symbiotic copepods and thereby challenge the validity of two terms: ”chalimus” and ”preadult”, that are only used with reference to the ontogeny of certain fish-parasitic copepods.

## 2. Materials and Methods

The principal publications which prompted us to propose changes in the terminology of caligid life-history studies were Anstensrud [9], Ferrari and Dahms [10], Venmathi Maran et al. [11], and Hamre et al. [12]. Those papers have indeed inspired us but they represented the traditional paradigm [9,11,12]. Ferrari and Dahms [10] opted for the reconciliatory terminology but without further discussion or explicit motion.

The introduced corrections in the accepted names of fishes were aided by the World Register of Marine Species [1].

We believe that the cited and analyzed scientific literature on the subject is complete for the past 40 years or so. We are confident to say that our literature search for other decades of the 19th and 20th centuries was also complete for the sources written in the Latin alphabet. We cannot exclude, however, that we missed some minor, unknown, and uncited sources written in a non-Latin script.

The names used for the developmental stages exhibit variability in the original literature (e.g., second chalimus, chalimus II, or chalimus 2). Therefore, in this paper, we will denote all the previously reported stages in a unified way, using Arabic numbers (e.g., chalimus 4). In the proposed new integrative nomenclature, we will use Roman numerals (e.g., copepodid V).

## 3. Early Studies on Caligid Ontogeny

A juvenile stage of a caligid copepod was first observed by Burmeister [13], who described a specimen collected from a mackerel captured off Helgoland in the North Sea. The copepod was relatively small and attached by “ein Fortsatz eigenthümlicher Art” [an extension of a peculiar kind] (p. 295, [13]). Burmeister described and illustrated this “extension” in detail as having a tripartite proximal part and a thread. Burmeister described his finding not only as a new species but he has also erected a new genus *Chalimus* for it. Krøyer [14] sustained the genus *Chalimus*, although not without hesitation, suggesting that it might be just a young *Caligus* and that further observations were necessary to confirm the situation. Milne-Edwards [15] repeated the information provided by Burmeister and Krøyer. Goodsir [16] observed a nauplius of a *Caligus*. Baird [17] described a nauplius of a *Caligus*, a chalimus stage, and its filament. He depicted the chalimus in dorsal view attached to an adult “*Caligus Mulleri*” [accepted as *Caligus curtus* Müller, 1785], and referred to the filament as a “rostrum”. He again treated *Chalimus* as a valid genus. Müller [18], observing the molt of a chalimus into an adult, was the first to prove that *Chalimus* is a juvenile stage of a *Caligus*. Subsequent authors, such as Hesse [19] and von Nordmann [20], accepted the findings of Müller. Gadd [21] reported and illustrated a chalimus 4? of *Caligus lacustris* Steenstrup et Lütken, 1861. Scott [22] described and depicted a nauplius and a chalimus stage of *Lepeophtheirus pectoralis* (Müller, 1776) and two chalimi of *Caligus rapax* Milne Edwards, 1840. Wilson [23] devoted more than 20 pages to the ontogeny of *Caligus rapax* and *Caligus bonito* Wilson, 1905 providing a wordy description and a selection of drawings. His narrative, however, was poorly structured and was not always consistent and/or complete. He also made some reference to *Lepeophtheirus pectoralis* and *Lepeophtheirus edwardsi* Wilson, 1905 and referred to the copepodid stage as the metanauplius. Gurney [24] described and illustrated two consecutive naupliar stages and the copepodid of *Lepeophtheirus thompsoni* Baird, 1850 and four (?) chalimus stages of *Lepeophtheirus* sp. from whiting. Unfortunately, the stages were not illustrated, so their identification is not possible. Similarly, as Wilson, Gurney was uncertain about the number of chalimus stages or even copepodid stages.

## 4. Advanced Research on Caligid Life Cycles

Early researchers, despite their determined attitude, were not always able to record all molts, observe all stages, and correctly interpret their findings. It is also difficult to discriminate between “early studies” and “advanced research”. One such “advanced” work was that of Russel [25], whose description and illustration of a new species *Caligus pageti* Russell, 1925, also covered the larval and juvenile stages. These included the nauplius and two (?) “metanauplius” (=copepodid) stages, the latter being followed by the chalimus, concerning which he wrote, “Among these there were three sizes, showing at least three moults.” (p. 615, [25]). The life cycle of the same copepod species was soon restudied by Argilas [26]. He reported the nauplius, with a series of intermediate states, the “cyclopoid stage” (=copepodid; secreting a long brown frontal cord), and the chalimus stage (undergoing numerous metamorphoses). He considered the copepodid’s “rostrum”, which he illustrated, to be homologous to the frontal filament of the chalimus. He also mentioned a chalimus structured like an adult but bearing a frontal cord. In 1934 Gurney [27] published an interesting paper that summarized previous ontogenetic studies on caligid copepods while also providing his own original contribution to this issue. Observing *Caligus centrodonti* Baird, 1850, he noted two nauplius stages, illustrated a molting nauplius 2, and observed a molting copepodid with extruded and attached frontal filament. In each subsequent molts Gurney observed that a “new portion” is added to the filament base. In “Stage III” he reported three bases of the filament. In “Stage IV” (male and female dealt with separately) he reported and illustrated four filament bases, lunulae, and a sternal furca. Confusingly, his illustrations referred to “chalimus” (1 through 4), whereas his descriptions referred to “stage” (I through IV). Nominally, the work of Gurney [27] constituted the first account presenting four chalimus stages, although the true identity of those stages may be debated and challenged. It now seems clear that Gurney [27], biased by his observation that the number of filament bases corresponds to the chalimus stage, missed or misinterpreted early chalimus stages. It is also evident that his “chalimus 4/stage IV” was in fact a young adult.

The first description of the life cycle of *Lepeophtheirus salmonis* (Krøyer, 1837) [now considered to consist of the two subspecies *L. salmonis salmonis* (Krøyer, 1837) and *L. salmonis oncorhynchi* Skern-Mauritzen, Torrissen et Glover, 2014] was published by White [28]. Despite its promising title, this was more of a preliminary study, in which the following developmental stages were reported: “metanauplius, (=copepodid) (p. 26, [24]), “first chalimus stage”, “second chalimus stage”, “several other chalimus moults”, “final chalimus stage”, and “numerous moults before the parasites attain their maximum size” (pp. 27–28, [28]). The “final chalimus stage” featured a marginal membrane and a filament. All stages were depicted in a dorsal view. The author was not able to obtain the nauplius.

White’s article was soon followed by the treatise of Heegaard [29], who appears to be one of the very few researchers who have purposely planned a career as an expert on parasitic copepods (personal communication of Z. Kabata to WP, 1987). In his book, Heegaard included a historical review of the previous studies on the biology of Caligidae, as well as original contributions including the description of the life cycle of *Caligus curtus*. The text, however, was verbose and the results were not separated from the discussion. Some parts are confusing, multi-threaded, and difficult to follow (e.g., pp. 39–40). Nevertheless, Heegaard’s [29] treatise is still the most extensive published account of caligid copepod biology and should be carefully (though in some aspects also critically) studied by students of copepodology. Heegaard included all details of his observations, even those that seemed insignificant. The presented life-cycle study included aquarium and in vitro observations of the behavior of the fish and copepods, the timing of events, and the external morphology and anatomy (including histology) of the copepod’s ontogenetic stages. Heegaard described in detail all the steps in the copepod’s development that he was able to observe and interpret: nauplius 1, nauplius 2, copepodid 1, copepodid 2, pupa, and 5 chalimi preceding the adult. Kabata [30] noticed that Heegaard’s “pupa” stage was comparable to chalimus 1 of other species. Copepodid 1 had a rostrum that was probably used for emerging from the second naupliar cuticle and copepodid 2 was attached to a fish by means of its frontal filament. It differed from copepodid 1 in having a new frontal filament already formed within its cephalothorax. The pupa, which was also attached to its host fish by a frontal filament and additionally had a full-sized new filament visible within its body, was evidently really a chalimus 1. Heegaard’s chalimus 2 was probably a chalimus 3 and his chalimus 3 was probably chalimus 4; It is likely that he missed chalimus 2. His chalimus 4 and chalimus 5, which were probably actually young adults, were both still attached to their host fish by a filament.

Kozikowska [31] studied the developmental stages of *Caligus lacustris*, the only freshwater representative of its genus, as part of a study on copepods parasitic on fishes in the estuary of the Odra/Oder River, mostly in the Polish part of the Szczecin Lagoon. The author also broadly reviewed historical references related to the collected copepods’ hitherto reported host fishes and geographical distribution. Kozikowska collected *C. lacustris* from a juvenile pikeperch, “*Lucioperca lucioperca*” [accepted as *Sander lucioperca* (Linnaeus, 1758)], captured in Wicko Lake. Having a total of 14 specimens, she described and illustrated the copepodid, chalimi 1, 2, and 3, and the adult female attached by a filament. The author was probably misled by the number of filament bases, so her “chalimus 3” was probably chalimus 4 judging by its morphological advancement and the filament base structure. Similarly, her “chalimus 2” was probably chalimus 3.

Lewis’s [32] work on the life cycle of *Lepeophtheirus dissimulatus* Wilson, 1905 represented a substantially more complex kind of ontogenetic study of these parasitic copepods. He not only illustrated the morphology of the successive stages but also plotted the basic morphometric data against time. His study describing and illustrating the nauplius 1, nauplius 2, copepodid, chalimus 1, chalimus 2, chalimus 3, chalimus 4, chalimus 5, chalimus 6, and adult was well organized and well structured. A special feature, presented by Lewis [32] for the first time and rarely followed in subsequent copepod life-cycle studies, was to show the ontogenetic changes of selected appendages. This approach dominated his description of the life cycle. No separate sections describing individual stages were provided; instead, the author focused his attention on the ontogenetic changes of the appendages. He also suggested that the filament can be re-attached. Ohtsuka et al. [33] and Venmathi Maran et al. [11] later revived his idea of counting the setae of the proximal segment of the antennule.

Hwa [34] studied the life history of *Caligus orientalis* Gusev, 1951. He collected developmental stages of the copepod from cultured “*Tilapia mossambica*” [accepted as *Oreochromis mossambicus* (Peters, 1852)] and determined that the life cycle consists of the following stages: nauplius 1, nauplius 2, copepodid, chalimus 1, chalimus 2, chalimus 3, chalimus 4, chalimus 5, and adult. A copepodid with an extruded filament was illustrated, as well as all other stages with details of their appendages and—something that must be emphasized—their frontal filament structure. Chalimus 5 had a marginal membrane and lunulae and its frontal filament had four extension lobes; therefore, we believe it was a young adult.

Izawa [35] studied the life cycle of *Caligus spinosus* Yamaguti, 1939 obtained from cultured yellowtail, *Seriola quinqueradiata* Temminck et Schlegel, 1845. In this important work, he distinguished, described, and illustrated the following stages: nauplius 1, nauplius 2, copepodid, chalimus 1, chalimus 2, chalimus 3, preadult 1, preadult 2, and adult. Both the first and the second preadult featured lunulae and a marginal membrane. One of the specimens of the first preadult had a frontal filament. This was the first paper to introduce the term ”preadult” to denote juvenile adult specimens that are not obligatorily attached to the host by a filament. The structure of the frontal filament of this particular species was totally different from that of the filaments of other described *Caligus* species. No regularity in this respect was observed in sequential stages and it appears that the extension elements of new stages were rod-like rather than lobe-like structures. It is likely that the two preadult stages were actually young adults. The author plotted body length and width for the individual stages and, quite importantly, showed that the lengths of chalimus 3 and preadult 1 do not overlap. This gap suggests that one stage, chalimus 4, was overlooked.

Another advanced study on caligid development was that of Voth [36], who wrote a doctoral thesis on the life history of *Lepeophtheirus hospitalis* Fraser, 1920. This thesis was not officially published, but it has been widely disseminated in paper as well as electronic form. The following stages were described and illustrated: nauplius 1, nauplius 2, copepodid, chalimus 1, chalimus 2, chalimus 3, chalimus 4, chalimus 5, chalimus 6, and adult. Even though Voth knew of Izawa’s paper [35], he decided not to use the term “preadult”. His “fifth chalimus” and “sixth chalimus” were quite advanced morphologically, both having a sternal furca and a marginal membrane, so they may have been young adults.

An important paper by Kabata [30] became a trend-setting publication. The author was already a leading specialist of copepods parasitic on fishes, and in the cited article he not only reported on the developmental stages of *Caligus clemensi* Parker et Margolis, 1964 but also analyzed and compared the hitherto described life cycles of caligid species and created a paradigm for an ontogenetic sequence consisting of four chalimi followed by two preadults. Although Kabata described only one preadult, he concluded that he must have missed the second one in his study. He admitted, however, that his “preadult” in general appearance differed from the adult only in having “a relatively poorly developed genital complex”. Kabata commented on the diversity of ontogenetic patterns in the published descriptions of known caligid life cycles, but he attributed the differences to “semantic misunderstandings”. Unfortunately, Kabata did not describe or adequately illustrate the proximal filament base. In a later review paper [8], Kabata further emphasized the existence of a “preadult” phase in caligid development.

Quite soon thereafter, Boxshall [37] published a paper on the developmental stages of *Lepeophtheirus pectoralis*. He described and illustrated the following stages: nauplius 1, nauplius 2, copepodid, chalimus 1, chalimus 2, chalimus 3, chalimus 4, preadult 1, preadult 2, and adult. In the copepodid, he observed a rostrum on the frontal plate. The male preadult 1 was attached to the host by a filament while also featuring a marginal membrane and sternal furca, and the preadult 2 had no filament. Both preadult stages were morphologically advanced and differed from the adults mainly in the body proportions.

In 1979 Caillet [38] wrote a doctoral thesis on the comparative biology of two species of parasitic copepods, one of which was *Caligus minimus* Otto, 1821. He described two nauplius stages, a copepodid, and five chalimus stages, but was not able to discriminate a separate preadult stage separated by molt. The copepodid had a rostrum as well as a filament visible within the cephalothorax. Chalimi 4 and 5 had lunulae, a sternal furca, an H-suture, and a marginal membrane and were attached to the host fish by a filament. Chalimus 3 showed primordia of the H-suture and thus was probably really chalimus 4. His chalimus 1 and 2 were probably really chalimus 2 and 3, respectively, and it is possible that he missed chalimus 1 altogether. The author dedicated several figure plates to a comparison of the ontogeny of individual appendages. Although fully aware of the “preadult” described earlier by authorities like Kabata, Caillet [38], he admitted that no preadult was observed in the life cycle of *Caligus minimus*.

The work of Wootten et al. [39] was an example of the acceptance of a 10-stage life cycle (including two preadult stages) for caligids based on the examples of *Lepeophtheirus salmonis* and *Caligus elongatus* on farmed salmonids.

In a dissertation, Ben Hassine [40] presented her studies on the life cycle of *Caligus pageti*. She reported two nauplius stages, one copepodid, four chalimi, one preadult, and the adult. Her chalimus 3 was probably really chalimus 4 because of the presence of an H-suture, a frontal filament with 3(?) lobes, and lunulae visible beneath the cuticle. Her chalimus 4 with a marginal membrane and functional lunulae was probably a young adult, and it was attached by a filament with 4(?) lobes. Misled by the standing paradigm, she apparently missed chalimus 2 because her chalimus 2 with a bilobate(?) proximal end of the filament looks like chalimus 3. Her preadult 1 was clearly a more advanced young adult without a frontal filament.

The end of the 1980s and the beginning of the 1990s marked the beginning of intensive sea lice studies. Aquaculture operations for Atlantic salmon not only began to increase in numbers, but also spread to regions nonnative to *Salmo salar* (e.g., Chile, British Columbia, Tasmania, and New Zealand [41]). In some countries, previously unreported sea lice species became problematic in the aquaculture of local fish species [42]. The losses attributed to sea lice were estimated to be millions of dollars [41]. Therefore, intensive efforts were focused on finding an effective remedy for sea lice. Unfortunately, the most effective organophosphate treatment methods were quickly criticized for their adverse environmental effects. This situation resulted in the allocation of research funds to study the biology of these parasitic crustaceans in the hope of finding alternative, non-chemical treatment methods. As a result, by the end of the 1980s and the beginning of the 1990s unprecedented numbers of papers on sea lice were published. In September 1992, the first big sea lice workshop with some 85 participants took place in Paris, France [43]. The topics discussed there included the life cycle stages, developmental factors, anatomy, behavior, epidemiology, and control of sea lice, with this last topic covering fallowing, chemotherapy, vaccination, biological control, and pathology. Around this time the most intensively targeted species were *Lepeophtheirus salmonis* and *Caligus elongatus* because of their particularly deleterious effects on salmon aquaculture.

Hogans and Trudeau [44] published a preliminary description of the life cycle of *Caligus elongatus*. They reported two naupliar stages, one copepodid, four chalimi, one preadult, and the adult, all nicely illustrated but unfortunately showing only the overall appearance of the individual stages. In all chalimus stages the frontal filament was surprisingly unnaturally short, and it supposedly became shorter in sequential stages. Their chalimus 2 was probably actually chalimus 3 because of its morphological advancement, developed H-suture, filament structure, and dramatic increase in size in relation to the preceding stage; consequently, their chalimus 3 was probably chalimus 4. Their chalimus 4 (featuring a marginal membrane) and “preadults” were definitely young adults representing two steps in morphological advancement. It is clear that these authors missed chalimus 2 altogether.

A fundamental and highly cited, but surprisingly poorly appreciated and/or understood, work was that of Anstensrud [9]. He observed that “during the ecdysis, all preadult and adult *Lepeophtheirus pectoralis* were observed attached to the host by a frontal filament.” (p. 271, [9]). “Within 1–2 h, moulting is completed. After another 2–3 h, the new exoskeleton has hardened, and the newly moulted individual detaches itself from the frontal filament and can move freely on the body surface of the host.” (pp. 271–272, [9]). What is also important, Anstensrud [9] illustrated the frontal organ of an adult female, which was very similar to the structure depicted by Piasecki and MacKinnon [45] for *C. elongatus* and by Ohtsuka et al. [46] for *C. undulatus* Shen et Li, 1959. This means that both the so-called preadult that up to then (and, unfortunately, also subsequently) had been reported in the life cycles of species of the genus *Lepeophtheirus*, and also the adults, need and use the filament as an indispensable element of their post-ecdysial period of life! The so-called preadults are in fact chalimi, and in light of Anstensrud’s [9] findings the concept of ”preadult” could no longer be sustained. Unfortunately, this brilliant copepod biologist died the same year in a tragic diving accident [47], and his important observations were effectively ignored for the next 32 years!

Bron et al. [48] studied the settlement and attachment of early stages of the salmon louse, *Lepeophtheirus salmonis*. This work was well illustrated with SEM micrographs. Describing the attachment process they stated, “Filament production must be rapidly followed by the moult to the first chalimus stage, since no copepodites were found attached by a filament.” (p. 203, [48]) They also noticed that the “basal plate” used to glue the filament to the fish stains differently than the filament stem. The latter “stained similarly to the rest of the exoskeleton, indicating that might be cuticular in origin”. Bron et al. [48] demonstrated the presence of an “axial duct” in the filament stem and described in detail the filament-producing apparatus. They suggested that the “axial duct” might deliver a cement substance to the distal end of the filament. The nature of the “filament duct” depicted on one of their SEM micrographs is, however, unclear.

At the beginning of the 1990s, when the intensity of the sea lice studies increased, Johnson and Albright [49,50] provided a description of the life cycle and all developmental stages of *Lepeophtheirus salmonis*. They distinguished the following stages: nauplius 1, nauplius 2, copepodid, chalimus 1, chalimus 2, chalimus 3, chalimus 4, preadult 1, preadult 2, and adult. The copepodid featured a rostrum and the preadult stages were quite advanced morphologically. They differed from the adults in their body proportions and some details of the appendage morphology.

Ogawa [51] studied the developmental stages of *Caligus longipedis* Bassett-Smith, 1898. The early stages were reared in the lab, whereas the more advanced stages were collected from their host. The author described and illustrated the nauplius 1, nauplius 2, copepodid, chalimus 1, chalimus 2, chalimus 3, chalimus 4, preadult, and adult. The preadult had lunulae and a marginal membrane and it was attached by a filament. The author stated that the preadult differed from the adult in having a thinner cuticle and a poorly developed genital complex; we interpret this stage as a young adult.

In the proceedings of the 1992 Paris Sea Lice conference [43], three papers dealt with life cycles and or developmental stages, namely Lin and Ho [52], Kim [53], and Schram [54]. Lin and Ho [52] studied the ontogeny of *Caligus epidemicus* Hewitt, 1971 parasitic on tilapia (*Oreochromis mossambicus*) cultured in brackish water. They observed and described a record high number of stages: nauplius 1, nauplius 2, copepodid, chalimus 1, chalimus 2, chalimus 3, chalimus 4, chalimus 5, chalimus 6, preadult, and adult. Unfortunately, the stages were not illustrated, and this account should, therefore, be considered preliminary. Kim [53] described and illustrated the nauplius 1, nauplius 2, copepodid, chalimus 1, chalimus 2, chalimus 3, chalimus 4, and adult of *Caligus punctatus* Shiino, 1955 collected from “*Chaenogobius castaneus*” [accepted as *Gymnogobius castaneus* (O’Shaughnessy, 1875)] in Korea. Some young adults were attached by a filament but Kim [53] refrained from discussing the number of developmental stages in the Caligidae. Schram [54] probably worked concurrently with but independently of Johnson and Albright [49,50] in another attempt to determine the life cycle of *Lepeophtheirus salmonis*. He was late in publishing, however, and his paper’s title (“Supplementary descriptions …”) acknowledged Johnson and Albright’s priority. Schram’s [54] description was nonetheless of high quality and his illustrations (including SEM) were supreme. Acknowledging the standing paradigm of Kabata [30], Schram listed the following stages: nauplius 1, nauplius 2, copepodid, chalimus 1, chalimus 2, chalimus 3, chalimus 4, preadult 1, preadult 2, and adult. He noted, however, that the two preadult stages differed from the adult only in the relative proportions of the genital complex.

Other important papers in the proceedings of the 1992 Paris Sea Lice conference [43] were those of Pike et al. [55,56] on the ultrastructure of the frontal filament in chalimus stages of *Caligus elongatus* and *Lepeophtheirus salmonis* from Atlantic salmon. Using TEM, they effectively described and compared the inner structure of the filament while, however, disregarding the changes in the filament structure of *Caligus elongatus* that occur during the course of its ontogeny. One of their papers [56] also contains some SEM micrographs, although Figure 3 in that work is either mislabeled or was misunderstood by the authors. It shows the proximal end of the filament of an adult *Caligus elongatus* but is labeled “chalimus 1”. A similar study was carried out concurrently by Piasecki and MacKinnon [45], but only in relation to the filament structure of *Caligus elongatus*. Their results are discussed in detail below.

Lin et al. [57] elaborated on their preliminary report [52] on the developmental stages of *Caligus epidemicus* parasitizing tilapia cultured in brackish water. They described the highest number of larval and juvenile stages amounting to 10: nauplius 1, nauplius 2, copepodid, chalimus 1, chalimus 2, chalimus 3, chalimus 4, chalimus 5, chalimus 6, preadult, and adult. This time they were hesitant and clearly stated that “no moult was observed between the preadult and the adult stage”. (p. 661, [57]) They illustrated a rostrum on the frontal plate of the copepodid. Chalimi 5 and 6 were advanced morphologically and had a marginal membrane and lunulae. The armature differences between these two stages were small. On the exopod of leg 4 “terminal inner seta increased greatly in length” between chalimus 4 and chalimus 5 (p. 681, [57]), and the ramus “becomes more elongated” between chalimus 5 and chalimus 6 (p. 681, [57]).

**Figure 1:**
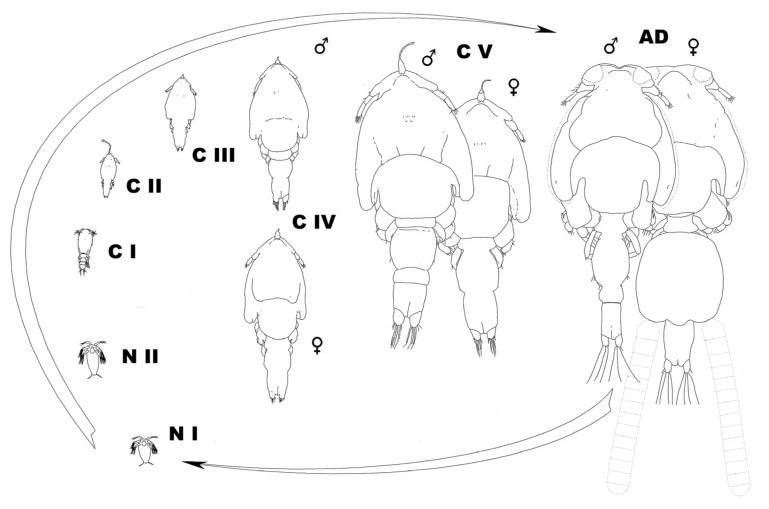
Diagrammatic representation of the life cycle of *Caligus elongatus*. Silhouettes of all stages are on the same scale. Stage numbers referring to the new interpretation of the present paper (with old nomenclature in the parentheses): N I = nauplius I, N II = nauplius II, C I = copepodid I, C II = copepodid II (chalimus 1), C III = copepodid III (chalimus 2), C IV = copepodid IV (chalimus 3), C V = copepodid V (chalimus 4), AD = adult. (Reprinted from Piasecki 1995 [64]; Piasecki^©^).

The life cycle of *Caligus elongatus*—later shown to be a collective name that has been applied to a number of separate genotypes and/or cryptic species [58,59,60,61]—was described by Piasecki and MacKinnon [62], and the morphology of its developmental stages by Piasecki [63]. Those observations, supported by those of Piasecki and MacKinnon [45], conclusively proved that no preadult is present in this species and that the cycle includes only the nauplius 1, nauplius 2, copepodid, chalimus 1, chalimus 2, chalimus 3, chalimus 4, and adult (Figure 1). The details were first presented in 1993 at the 5th International Conference on Copepoda in Baltimore, MD, USA.

Lin et al. [65] studied the development of supposed “*Caligus multispinosus* Shen, 1957" from black sea bream, *Acanthopagrus schlegelii* (Bleeker, 1854), cultured in Taiwan. As was later demonstrated by Ho et. al. [66], the copepod was actually *Caligus rotundigenitalis* Yü, 1933. Lin et al. [65] identified two nauplii, one copepodid, four chalimus stages, one preadult, and the adult. The early copepodid was illustrated with a rostrum and the “advanced” copepodid was illustrated in the process of molting. The proximal part of the frontal filament was well described and illustrated, and it was consistent with the pattern described by Piasecki and MacKinnon [45]. The preadult was attached by a filament and was rather advanced morphologically, having a marginal membrane, lunulae, and a sternal furca; this preadult was, therefore, actually a young adult.

Gonzalez-Alanis et al. [67] described the morphogenesis of the frontal filament in the salmon louse *Lepeophtheirus salmonis*. This study was a continuation and a progressive development of the research reported by Bron et al. [48]. González and Carvajal [68] studied the life cycle of *Caligus rogercresseyi* infecting cultured salmonids in Chile. They found and described two nauplii, one copepodid, four chalimus stages, and the adult. No preadult stage was observed. Unfortunately, the developmental stages were not illustrated in detail. The development of the frontal filament followed the pattern described by Piasecki and MacKinnon [45].

In their book “Sea lice of Taiwan”, Ho and Lin [69] repeated the information on the ontogeny of *Caligus epidemicus* and *C. rotundigenitalis* provided in the two earlier works by Lin et al. [57,65]. They discussed not only the morphology of the instars but also emphasized the different structures of the frontal filament in *Caligus epidemicus*, arguing that in this particular species, each stage has a different frontal filament and at each molt, a new filament must be formed. It is worth mentioning that a frontal filament with a similar rod-like structure was observed in *Caligus spinosus* by Izawa [35].

In their monograph on the post-embryonic development of the Copepoda, Ferrari and Dahms [10] wrote, “chalimus 1 of caligid-like copepods resembles CII of other copepods in the number and kind of somites: a cephalon with five limbs, six thoracic somites, and a posterior abdominal somite.” (p. 55, [10]). They explicitly stated that “the four chalimus stages correspond to the second to fifth copepodid stages.” (p. 217, [10]).

Ohtsuka et al. [33] studied the developmental stages and growth of “*Pseudocaligus fugu*” (accepted as *Caligus fugu* Yamaguti, 1936). They described and illustrated two nauplii, one copepodid, four chalimus stages, and the adult. They reviewed all known life cycles including 11 full life cycles for *Caligus* and 4 for *Lepeophtheirus*. They also summarized and discussed the hitherto published information on the frontal filament and emphasized the importance of the setation of the antennule’s proximal segment for discriminating the juvenile stages.

Madinabeitia and Nagasawa [70] studied the chalimus stages, but not the nauplius or copepodid stages, of *Caligus latigenitalis* Shiino, 1954 parasitic on blackhead seabream, *Acanthopagrus schlegelii* (Bleeker, 1854), from Japan. They introduced the term ”semaphoront” to denote a functional (i.e., morphologically different) stage that is not necessarily separated by a molt. Within the copepodid stage, they distinguished two semaphoronts (the infective copepodid and the chalimus copepodid), which were followed by four chalimus stages (=semaphoronts) and the adult stage (with two semaphoronts: the chalimus adult and the mobile adult). They amended the definition of ”chalimus”, distinguishing between the stage and the semaphoront [71]. Accordingly, a ”chalimus” may be either a post-naupliar stage corresponding to copepodids II through V [10] or a semaphoront with a frontal filament used for attachment to the host.

Venmathi Maran et al. [11] described the life cycle of *Lepeophtheirus elegans*. They determined that the traditionally recognized chalimus 1 and 2 should be merged into a single stage—chalimus 1—and similarly that chalimus 3 and 4 represent a single stage—chalimus 2. Consequently, they listed the following stages in the life history: nauplius 1, nauplius 2, copepodid, chalimus 1, chalimus 2, preadult 1, preadult 2, and adult. Like Ohtsuka et al. [33], Venmathi Maran et al. [11] used the number of setae on the proximal segment of the antennule to differentiate between the post-naupliar stages. Their new model of the caligid life cycle reconciled the sequences of development of *Caligus* and *Lepeophtheirus* for the first time and will be very important for the final conclusions of the present paper.

Hamre et al. [12], probably working concurrently with and independently of Venmathi Maran et al. [11], studied *Lepeophtheirus salmonis* and achieved comparable results. They also observed chalimus 1 (merging the former chalimus 1 and 2) and chalimus 2 (merging the former chalimus 3 and 4). They emphasized that their study was the first to be based on direct observation of molts and/or shed exuviae of all the developmental stages. The “external lamina” of the frontal filament (as defined by Bron et al. [48]), which had been supposed to be a remnant of the shed exuvium of chalimus 1, is in fact an extension of the chalimus’s cuticle. Hamre et al.’s [12] direct molt observations were backed up by the morphometric clustering analysis. Their study marked an important step in the advancement of the methodology for life-cycle research on caligid copepods. The same team developed this methodology further, as published by Eichner et al. [72], to study instar growth and molt increments in the chalimus stages of *Lepeophtheirus salmonis* and to provide additional important observations regarding the frontal filament.

Khoa et al. [73] studied the life cycle of the species identified as *Caligus minimus* infecting seabass, *Lates calcarifer* (Bloch, 1790), in floating cage culture. They recognized eight developmental stages: nauplius 1, nauplius 2, copepodid, chalimus 1, chalimus 2, chalimus 3, chalimus 4, preadult, and adult. The authors were apparently unaware of the unpublished thesis of Caillet [38], who had studied the ontogeny of the same species. The authors provided photographs of the various stages but no morphological details, and some of the stages appear to be mislabeled.

Hamre et al. [74] monitored the development of parasitic stages of *Lepeophtheirus salmonis* reared in temperatures ranging from 3 to 24 °C.

Hemmingsen et al. [75] provided a comprehensive review of *Caligus elongatus* and other sea lice of the genus *Caligus* infecting farmed salmonids. They summarized the available literature but for unknown reasons perpetuated the preliminary and erroneous results of Hogans and Trudeau [44], especially by reproducing their figures with unrealistic frontal filaments.

Under controlled conditions, Bravo et al. [42] compared the life cycles of *Lepeophtheirus mugiloidis* Villalba et Duran, 1986 and *Caligus rogercresseyi*, parasites of the Patagonian blenny *Eleginops maclovinus* (Cuvier, 1830). They reported the existence of nauplius 1, nauplius 2, copepodid, chalimus 1, chalimus 2, preadult 1, preadult 2, and the adult in *L. mugiloidis* and nauplius1, nauplius 2, copepodid, chalimus 1, chalimus 2, chalimus 3, chalimus 4, and the adult in *C. rogercresseyi*. This paper was very brief, and no details of the developmental stages were provided.

One of the most recent papers on this subject was that of Jeong et al. [76], who quantified key parameters related to the life cycle of *Caligus rogercresseyi* and reported a developmental sequence of nauplius1, nauplius 2, copepodid, chalimus 1, chalimus 2, chalimus 3, chalimus 4, and adult.

Table 1 and Table 2 summarize the main morphological characters of the developmental stages of *Lepeophtheirus* and *Caligus*, and Table 3 provides a summary of “advanced” life cycle studies.

## 5. Reports on Individual Developmental Stages

A number of people have published papers concerning individual developmental stages of caligids. Among them was Markewitsch [77], who found and illustrated (in dorsal view) a “metanauplius” (=copepodid) and a “chalimus” (probably chalimus 3) of *Caligus lacustris* infecting many different fish species in the Caspian Sea. While studying fish-parasitic copepods of the Gulf of Mexico, Bere [78] found, described, and illustrated chalimus stages of a newly discovered species, *Caligus praetextus* Bere, 1936, which had been collected from the tail of a hogfish, *Orthopristis chrysoptera* (Linnaeus, 1766). Among Bere’s “chalimus a”, “chalimus b”, and “chalimus c”, the latter two might represent either chalimus 4 or young adults still attached by a very short and thick filament; unfortunately, the structure of the filament’s proximal end was not illustrated. Gnanamuthu [79] described sex differences in the advanced chalimus stages (possibly including young adults) of *Caligus polycanthi* Gnanamuthu, 1950 parasitic on “*Balistes maculatus*” [accepted as *Canthidermis maculata* (Bloch, 1786)] from Madras, India. His drawings were quite superficial and the filament structure was not shown. Kaj [80] found chalimus stages of *Caligus lacustris* on *Alburnus alburnus* (Linnaeus, 1758) from Górzyń Lake, Poznań Voivodship, Poland. His description was only general, and his drawings do not permit stage identification. Hewitt [81] described a new species, *Caligus epidemicus* Hewitt, 1971, while also covering some of its developmental stages, which he labeled as A, B, C, D, and E. Stage A was the copepodid. Stages B and C had lunulae visible beneath the cuticle, so they probably represented chalimus 4. Stage D was a young adult (probably male) with a filament, and stage E was an adult (female) without a filament. The author illustrated not only the respective habiti in dorsal view, but also compared the morphology of the appendages of stages A, B, C, and D. Hewitt’s descriptions and images reveal very little about the nature and structure of the frontal filament. Lopez [82] incubated egg-strings of “*Lepeophtheirus kareii*” (accepted as *Lepeophtheirus hospitalis* Fraser, 1920, the same species that Voth [36] studied) and managed to obtain two naupliar stages and the copepodid. Lopez documented their general appearance in photographs and their appendages in microscopic drawings. Johannessen [83] studied the early stages of *Lepeophtheirus salmonis*, describing and illustrating nauplius 1 and 2 and only briefly mentioning the copepodid, providing its length and width. Pike et al. [55] studied the naupliar stages and the copepodid of *Caligus elongatus* in relation to temperature. Izawa [84] described the copepodid, chalimus 2, and chalimus 4 of *Caligus latigenitalis* Shiino, 1954 parasitic on black sea bream, *Acanthopagrus schlegelii*, from Japan. The filament was reported as having a structure similar to that of *Caligus elongatus* as described by Piasecki and MacKinnon [45]. Izawa [84] also contended that the preadult described by some authors was not a true instar separated by a molt but simply a young adult. Jones [85] studied *Caligus patulus* Wilson, 1937 from a fish farm in the Philippines. He found the copepodid, chalimus 2, two preadult stages, and the adult female and male. Both of his preadult stages had lunulae, a marginal membrane, and a sternal furca, so they were probably young adults. Eichner et al. [72] studied instar growth and molt increments in chalimi of *Lepeophtheirus salmonis* and provided important observations regarding the frontal filament (see Section 7 below). Ohtsuka et al. [46] reported chalimus 3, chalimus 4, and the ovigerous female of *Caligus undulatus*. The ovigerous female was attached to its host fish by a frontal filament. Montory et al. [86] studied the early development of *Caligus rogercresseyi* under combined salinity and temperature gradients and recognized nauplius 1, nauplius 2, and the copepodid.

## 6. Frontal Filament in Species of *Caligus*

**Figure 2:**
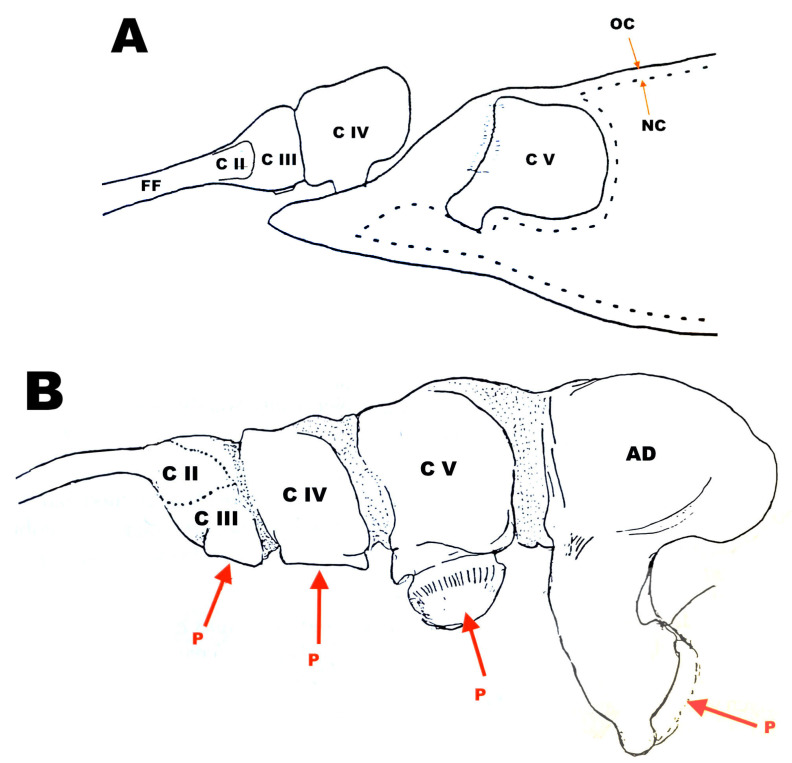
(**A**) Hypothetical, diagrammatic representation of the frontal filament extension lobes in a sagittal section of a pre-molt copepodid IV (chalimus 3) of *Caligus elongatus*; lateral view of the anterior part of the body. (**B**) Diagrammatic representation of the proximal part of the frontal filament in an adult of *Caligus elongatus*; lateral view. P denotes connecting pads of the consecutive stages. Abbreviations: C II = copepodid II (ch1), C III = copepodid III (ch2), C IV = copepodid IV (ch3), C V = copepodid V (ch4), AD = adult; FF = frontal filament shaft; OC = old cuticle, NC = new cuticle.

We usually understand the term ”chalimus” to denote a juvenile copepod stage that is attached to its host by a frontal filament. To define it better, however, we need to describe this structure in detail stage-by-stage. In most cases, our understanding of copepod juveniles attached permanently by a filament (or cord, thread, or tether) is overly simplified and limited to selected stages only. In reality, it is not that straightforward. A good example is *Caligus elongatus*, studied by Piasecki and MacKinnon [45,62] and Piasecki [63]. According to those sources (and WP’s unpublished information), the first traces of the frontal filament appear within the cephalothorax of older copepodids in the form of irregular particles of a dark-staining substance. During the next few hours, the filament becomes more developed and elongated. It is very likely that the filament is produced at the bottom of the cuticular pocket that extends from the frontal area to the center of the cephalothorax. The filament’s distal end is permanently attached to the fish, and the proximal end is the filament’s base. When the copepodid contacts the fish, the cuticular pocket evaginates in such a way that the copepodid carries the filament’s proximal end (the evaginated cuticular pocket) on its frontal area and the distal end of the filament becomes attached to the host [63]. This activity will be discussed in more detail below. The original filament will be “inherited” by all subsequent stages including the adult. The frontal organ, which is responsible for secreting the filament and its extension lobes, is originally (at the copepodid stage) located at the bottom of a cuticular pocket and migrates to the frontal area, where it remains in all subsequent stages. Each of the consecutive stages modifies the proximal end of the filament. Namely, before a molt the new instar prepares a filament extension lobe beneath its cuticle. During the molt, the old cuticle remains attached to the filament’s base. The newly emerging stage leaves the old cuticle behind, but its first action is to secure its own attachment to the host. To do this, it glues the new extension lobe, which is firmly attached to the frontal organ, to the filament base. The old cuticle eventually wears away and detaches, but its remnants are sometimes visible, attached to the connection pads of the previous stage’s filament extension lobe (Figure 2A).

The number of filament-base elements is directly related to the numbering of the chalimus stages; however, in chalimus 2, only one element of this base is visible; in chalimus 3, two such elements; in chalimus 4, three; and in the young adult, four. This increment in the number of the elements of the proximal end of the filament was understood literally by some researchers who believed, for example, that three elements identified the stage as chalimus 3 (etc.) This is because the smallest and earliest element belonging to chalimus 1, including its attachment pad, becomes completely engulfed by the element added by chalimus 2 and is thus completely obscured. Each subsequent filament extension piece is slightly larger than the previous one. Disregarding the engulfed element, the filament base has a single, semi-triangular lobe in chalimus 2, a bipartite lobe in chalimus 3, a tripartite lobe in chalimus 4, and finally a four-lobed base in the young adult (Figure 2B).

It is evident from the observations of Piasecki and MacKinnon [45] presented above that a frontal filament is possessed by all post-naupliar stages, namely the chalimus 1, chalimus 2, chalimus 3, chalimus 4, and adult. This means that the existing definition of ”chalimus” is not consistent, because of the arbitrary elimination of the copepodid and the adult from the concept. Cases of a copepodid attached by a filament, as has been reported by many authors, will be discussed below.

According to Piasecki and MacKinnon [45,62], adults need the filament to complete their last molt (chalimus to adult). Those authors also reported that ovigerous females were still attached by the filament. Also important, in conjunction with this last molt the filament receives its final lobe. If we stick to an unqualified definition of ”chalimus”, we would need to refer to the adult as chalimus 5. Also, the advanced (attached) copepodid appears to be attached by a filament. Therefore, we must regard the copepodid as a chalimus 1, and then the adult female still attached by a filament as chalimus 6? Remember that Izawa [35] illustrated the first preadult of *Caligus spinosus* as still being attached by a filament. This first preadult seems to be homologous with chalimus 5 of Piasecki and MacKinnon [45] and also with the adult of Piasecki [63]. Non-filament-mediated attachment of newly molted adults (preadults?) or any other stage seems impossible because of the soft post-molt cuticle. We believe that the condition of being attached by a filament does not define the stage but is rather a functional characteristic of several developmental stages, which are really copepodids in integrative terminology.

The filament structure of *Caligus epidemicus* described by Lin et al. [57] is totally different from that described for *C. elongatus* by Piasecki and MacKinnon [45], although very similar to that of *C. spinosus* reported by Izawa [35]. The differences are so profound that the different filament structures could potentially serve as important taxonomic traits. The extension element in *C. epidemicus* is rod-like and very elongate, so much so that it might be used as either an extension lobe or a brand-new filament if something goes wrong. Lin et al. [57] believed that only chalimus 4, not every post-naupliar stage as in *C. elongatus*, reuses the old filament of the preceding stage. The filaments of *C. spinosus* and *C. epidemicus* should be restudied with a focus on the consistency of the filament’s structure in successive stages.

## 7. Frontal Filament in Species of *Lepeophtheirus*

The filament in species of the genus *Lepeophtheirus* has a different structure from that in species of *Caligus*. Also, the strategy of attachment, its details, and changes during the sequence of developmental stages seems to be different. The filament is relatively short and stout, and no modification of its proximal end is observed during ontogeny [49]. The first comprehensive study on this subject was that of Bron et al. [48]. Using light microscopy for histological observations and a scanning electron microscope (SEM) to obtain external views of the structures, they determined that the filament consists of a fibrous stem with an axial duct and that the basal plate is attached to the epithelial basement membrane of the host fish. The filament is covered by “external lamina” supposedly continuous with the integument of the chalimus. The basal plate is formed from a secretion produced in the cephalothorax by the A-group gland cells they discovered; the secretion is delivered to the distal portion of the stem through the axial duct. B- and C-group gland cells were also defined, but their function was not characterized. The spatial locations of the three groups of gland cells were presented in diagrams.

Pike et al. [56] soon published a set of SEM and TEM observations including five micrographs of the filament of *Lepeophtheirus salmonis*, but they were rather hesitant to interpret them in detail. They did state, however, that the filament of *Lepeophtheirus salmonis* is an integral part of the chalimus body.

**Figure 3:**
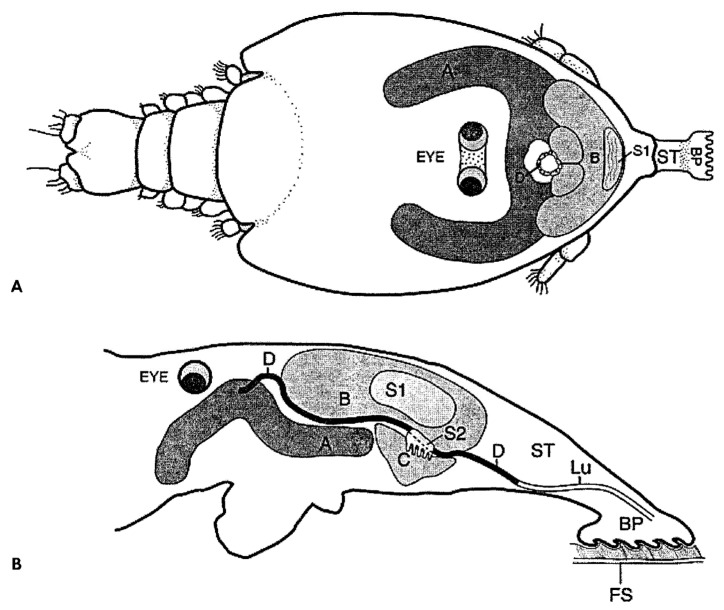
Diagrammatic representation and the arrangement of cell groups A, B, and C responsible for the secretion of the frontal filament and internal filament material S1, S2, and duct D, in chalimus of *Lepeophtheirus salmonis* at 7 dpi. Reproduced from Gonzalez-Alanis et al. [67] under the permission of the Journal of Parasitology. (**A**) Dorsal view; (**B**) Sagittal view; BP, basal plate; FF, frontal filament; FS, fish scale (Other abbreviations were not explained by [67].

Gonzalez-Alanis et al. [67] used high-resolution light microscopy to study histological sections of juvenile *Lepeophtheirus salmonis* and proposed the following sequence of events in filament development. First, the B-groups cells release secretion 1 (S1) (substance 1?), which forms the basal plate. Then the C-group cells produce S2, which becomes the stem of the external frontal filament. The A-cells secrete a glue-like substance, delivered to the distal part of the frontal filament through the axial duct. In pre-molt specimens, S1 and S2 are tightly enclosed within a cuticular invagination, first described by Bron et al. [48] as the “external lamina”. The spatial location of the three groups of gland cells was shown in very informative diagrams (Figure 3). Most importantly, Gonzalez-Alanis et al. [67] provided strong evidence that *Lepeophtheirus salmonis* produces a brand-new filament with each molt.

Anstensrud [9] determined that the frontal filament is present in each post-molt stage of *Lepeophtheirus pectoralis*, including the adults. This is very important for understanding the conclusions of the present paper. Eichner et al. [72] not only studied the growth and molt increments in chalimi of *Lepeophtheirus salmonis* but also made important observations on the frontal filament. They stated that Hamre et al. [12] had observed a frontal filament on the exuvium of chalimus 1. This statement is not precisely correct because it is evident from Figure 4 in [12] that the exuvium featured only the cuticular sheath of the filament, that is, Bron et al.’s [48] “external lamina”. Although Eichner et al. (71) observed no such structure in the exuvium of chalimus 2, Figure 4 in [12] apparently shows that the exuvium of this stage does indeed have an “external lamina”. Eichner et al. [72] also noticed that filaments are visible inside pre-molt juveniles. Based on other studies [9,49,67] as well as their own, they stated that “a total of five separate filaments are formed during the *L. salmonis* life cycle, that is, the filaments produced prior to molting by the copepodid, chalimus 1, chalimus 2, preadult 1 and preadult 2.” It is very possible that the “external lamina” does not occur in preadult 1 and 2, nor the adult, during their period of temporary attachment by the filament. The “external lamina” is a continuation of the integument, and it would be unlikely for such a structure to be discarded long before the actual molt (which does not occur in the adult). If it is present in preadults, we would expect to see its remnants around the frontal organ, but no such remnants are visible in Anstensrud’s [9] figures.

## 8. “Copepodid Attached by the Frontal Filament”?

In *Caligus* life cycles, a “copepodid attached by the frontal filament” has been observed and illustrated by Gurney [27], Hwa [34], Hogans and Trudeau [44], Piasecki [63], and Khoa et al. [73]. Such reports may prove deceptive when the course of events is analyzed carefully. For example, Izawa [35] stated that chalimus 1 is the stage that first attaches to the host by the filament; but this is also an oversimplification. It is quite reasonable to believe that alternating actions of the clawed appendages and the frontal filament are needed for securing the attachment of consecutive instars in the life history of caligid copepods. We interpret the course of events during the post-naupliar phase of the ontogeny as follows:

○ The copepodid finds a suitable place on the surface of the host and firmly attaches there using the claws of the antennae and the maxillipeds;
○ Through intensive scraping by some appendages it removes a portion of the host’s epithelium, thereby exposing a suitable place for permanent attachment;
○ Molting starts with a rupture of the copepod’s larval cuticle in the frontal area;
○ The cuticular pocket containing fully formed frontal filament, previously visible within the cephalothorax, evaginates to become a frontal extremity;
○ The filament is released and its proximal end becomes firmly attached to the tip of the frontal extremity;
○ The distal end of the filament is glued to the previously exposed part of the host’s body;
○ After the hardening of the filament has assured that the attachment to the host is secure, the organism continues its molting process;
○ The next stage—copepodid II (chalimus 1)—withdraws from the copepodid I exuvium and hangs from the host, attached only by the frontal filament. The exuvium itself is discarded.

**Figure 4:**
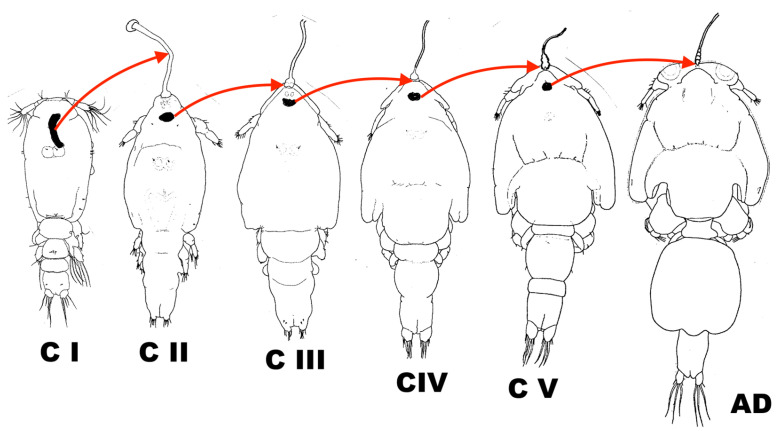
The sequence of events in the formation of the frontal filament in consecutive developmental stages of *Caligus elongatus*. In pre-molt specimens, extension lobes (black) are visible beneath the old cuticle (in the copepodid it is the future frontal filament). Abbreviations: C I = copepodid I, C II = copepodid II (ch1), C III = copepodid III (ch2), C IV = copepodid IV (ch3), C V = copepodid V (ch4), AD = adult. Silhouettes of the stages are based on those from Piasecki 1995 [64] (Piasecki^©^).

An alternative course of events whereby the copepodid is already attached to its host by a filament is rather unlikely. This is because every molt is preceded by the formation of the next-stage filament element beneath the cuticle (Figure 4). Copepodid I produces the entire frontal filament. Copepodid II (chalimus 1) produces the extension lobe of copepodid III (chalimus 2), copepodid III (chalimus 2) produces the extension lobe of copepodid IV (chalimus 3), etc. If copepodid I was already attached by the filament it would also need to produce the extension lobe of the next stage, something which has never been observed and it is unlikely to occur considering the extremely short time before the attachment of the original filament and the first occurrence of copepodid II (chalimus 1). The so-called “copepodid attached by a filament” is definitely not a stage but it may be treated as a “semaphoront” representing a copepodid I in the process of molting into a copepodid II (chalimus 1).

## 9. The Concept of ”Preadult”

The term ‘preadult’ was first introduced by Izawa [35], who distinguished two nauplii, a copepodid, three chalimi, and two preadults in the life cycle of *Caligus spinosus*. The preadults had lunulae, a marginal membrane, and a sternal furca. Some specimens of preadult 1 were even attached by a frontal filament. The preadults differed from the adult in their body proportions, mainly those of the genital complex. While the body lengths of the preadult 1 and adult did not overlap, the mean values for carapace length of the two preadult stages were similar (0.96/0.96 mm vs. 1.19/1.16 mm for females/males). Izawa doubted whether the preadult 2 and adult were really distinct stages.

The concept of the ”preadult” became institutionalized in the wake of Kabata [30], who suggested that regardless of the genus, the caligid life cycle should contain two nauplii, a copepodid, four chalimi, and two preadults. Even though Kabata himself observed only one preadult, his model became the standing paradigm for the next 50 years. The term ‘preadult’ has been used to denote young adults of species belonging to both *Caligus* [8,39,40,41,44,51,52,57,65,85,87] and *Lepeophtheirus* [8,37,39,41,49,50,54,87,88,89,90,91,92,93,94], and very few researchers have admitted to observing no preadult [38,45,51,62,63]. Ho and Lin [69] nonetheless concluded that the so-called preadults described in *Caligus* species are freshly molted, young adults, and some subsequent life cycle studies did not report preadult stages for *Caligus* [33,70]. Only recently was the term ”preadult” redefined to denote stages separated by one or more molts from the adult [11]. By this definition, the preadults (1 and 2) of *Lepeophtheirus elegans* [11] and *Lepeophtheirus salmonis* [12] were revealed as homologous, respectively, with chalimus 3 and 4 of *Caligus* and copepodid IV and V of all podoplean copepods [8,10].

Preadults 1 and 2 reported by Venmathi Maran et al. [11] and Hamre et al. [12] were in fact advanced juvenile stages, most probably already using their frontal filament within hours after a molt. Such a phenomenon was observed by Anstensrud [9], and it is very likely that this is a common pattern among *Lepeophtheirus* spp. Use of a filament makes them chalimi. If we also consider the adult’s temporary use of the filament in *Caligus* and *Lepeophtheirus*, we might have to extend the term ”chalimus” to the adult as well. We believe that no molt in caligid ontogeny occurring on the host is possible without (at least) temporary attachment by a filament. In view of the above, it would be difficult to define preadult explicitly.

## 10. Recapitulation

**Figure 5:**
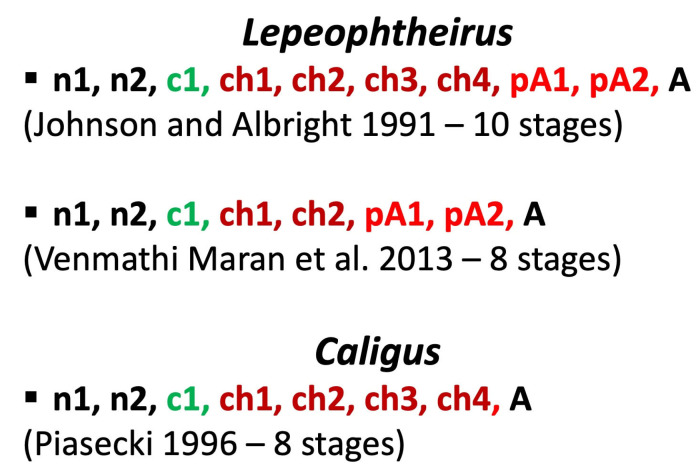
Alternative terminological sequences in the development of copepods of the family Caligidae based on examples from the genera *Lepeophtheirus* and *Caligus*. Abbreviations: n1 = nauplius 1, n2 = nauplius 2, c1 = copepodid 1, ch1 = chalimus 1, ch2 = chalimus 2, ch3 = chalimus 3, ch4 = chalimus 4, A = adult.

**Figure 6:**
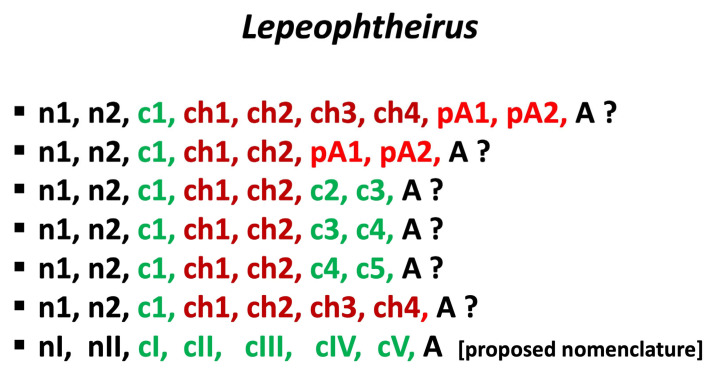
Alternative terminologies for ontogenetic sequences in the development of copepods representing the family Caligidae. The last row (using Roman numerals) is the proposed new terminology. Abbreviations: n1 = nauplius 1, n2 = nauplius 2, c1 = copepodid 1, ch1 = chalimus 1, ch2 = chalimus 2, ch3 = chalimus 3, ch4 = chalimus 4, c2 = copepodid 2, c3 = copepodid 3, c4 = copepodid 4, c5 = copepodid 5, A = adult, nI = nauplius I, nII = nauplius II, cI = copepodid 1, cII = copepodid II, cIII = copepodid III, cIV = copepodid IV, cV = copepodid V.

The preadult stage reported in species of the genus *Caligus* [30,35,40,44,51,57,65] turns out to be young adults mostly differing in the proportions of the genial complex (Table 3). Such preadults have all the morphological attributes of the adult (e.g., lunulae, marginal membrane, sternal furca, H-suture) and are not separated by a molt from the adult. In the genus *Lepeophtheirus*; however, the so-called preadults are indeed separated by a molt from the adult [11,12]. As was noted above, several authors have demonstrated that the four preceding chalimus stages in this genus should be merged into only two. The *Lepeophtheirus* life-cycle thus became reconciled with that of *Caligus* in terms of the number of post-naupliar stages (Figure 5), but not in terms of their terminology. Venmathi Maran et al. [11] and Hamre et al. [12] for some reason did not reconcile the two life cycles completely. Although they both cited Anstensrud [9], they were apparently deceived by the notion that preadults and adults have only a “temporary filament”. The apparent actual similarities between the life cycles of *Lepeophtheirus* and *Caligus* were obscured by semantic differences. If we insist that preadult 1 and 2 are not homologous with chalimus 3 and 4, respectively, then what are they? A chalimus is a copepodid attached by a filament. If it loses the filament or uses it only temporarily, does it become a copepodid again? If so, the life cycle of *Lepeophtheirus* spp. should be amended to contain only two real chalimi. We believe, to the contrary, that the validity of the term ”preadult” cannot be defended any longer and that the life cycle of both *Caligus* and *Lepeophtheirus* includes a nauplius 1, nauplius 2, copepodid 1, chalimus 1, chalimus 2, copepodid 4, copepodid 5, and adult. This still does not represent a full reconciliation of the two life cycle patterns (Figure 6). Why should chalimus 2 be followed by copepodid 4? And what has happened to “stage 3”? The best solution might be to abandon the term ”chalimus” altogether for life-history stages of these copepods. It might still remain in use by field researchers for making practical observations, but only as a “semaphoront” [70] because the actual “stage” cannot be determined in the field. Used as a semaphoront, the term ”chalimus” may refer to any filament-bearing stage from the copepodid to the adult (inclusive), but such use cannot be easily interpreted and converted to precise life-cycle stages.

Kabata [8] wrote “The life cycles of copepods belonging to Gymnoplea, which comprise only the almost exclusively free-living Calanoida, consist of 12 stages other than adult: six nauplii and six copepodids. In contrast, the free-living Podoplea, ancestors of the parasitic copepods, often have only five nauplius stages (although that number may be reduced) and six copepodid stages. Since parasitic copepods descended from podoplean ancestors, they can be expected to have not more, possibly fewer, than five nauplius stages.” (p. 20, [8]). Also “Remembering that our copepods have descended from the free-living Podoplea, we would expect that the species least modified by their pursuit of a parasitic way of life would have life cycles differing only slightly from those of their ancestors.” (p. 21, [8]).

If the number of copepodid stages in Caligidae is (almost) consistent with that of the podoplean ancestors, why the number of naupliar stages is so much reduced? The answer was suggested by Pedaschenko [95] who observed exuviae within the egg of *Lernaeocera branchialis* (Linnaeus, 1767). This phenomenon should be studied further, but if proven, it might explain why there are only two naupliar stages are in caligid life cycles. A further reduction of the naupliar phase can be observed in fish-parasitic copepods of the family Lernaeopodidae [96,97], in with only single nauplius stage that hatches from the egg and is immediately ready to molt into the copepodid.

As we mentioned earlier, Ferrari and Dahms [10] explicitly stated that “the four chalimus stages correspond to the second to fifth copepodid stages.” Consequently, we believe that the terms ”preadult” and ”chalimus” are no longer justified in studies on the biology of caligid copepods as they only contribute to confusion and should be abandoned. We suggest that copepods of the family Caligidae be deemed to have the following stages in their life cycle: nauplius I, nauplius II, copepodid I (infective), copepodid II (chalimus 1), copepodid III (chalimus 2), copepodid IV (chalimus 3), copepodid V (chalimus 4), and adult. An alternative, practical terminology may also contain an indication (F) of the presence of the frontal filament: nauplius I, nauplius II, copepodid I, copepodid II/F, copepodid III/F, copepodid IV/F, copepodid V/F, and the adult. Alternatively, a lower case “f” may be used for those stages where the filament is only temporary. For example, "adult f" means young one with a frontal filament.

This is a polemical paper, and our intention is to spark a discussion on this terminological problem. The general concept presented here may be extended to the life cycle of other siphonostomatoid copepods. According to Boxshall and Halsey [7], the following families are known to have “chalimus” stages with a frontal filament: Cecropidae, Lernaeopodidae, Nicothidae, and Pennellidae. Considering the phylogenetic placement of the Caligidae among other caligiforms [98], the families Dissonidae, Pandaridae, Sphyriidae, and Trebidae may also have “chalimi”. If so, our reinterpretation of the developmental stages can be extended to all these families. According to a recent molecular phylogenetic analysis on the order Siphonostomatoida [99], the family Nicothidae, which may not be monophyletic, seems to have developed frontal filaments independently of the caligiform group. A final hypothesis, that siphonostomatoids other than caligiforms and “nicothoids” might have secondarily lost the frontal filament cannot be ruled out.

## Figures and Tables

**Table 1:** Comparison of diagnostic features of developmental stages of *Caligus elongatus* (based on Piasecki [63]).

	Copepodid I	Copepodid II (Ch 1)	Copepodid III (Ch 2)	Copepodid IV (Ch 3)	Copepodid V (Ch 4)	Adult
Filament	1-lobed	1-lobed	1-lobed *	2-lobed	3-lobed	4-lobed
3rd leg	Vestigial	Vestigial	Biramous	Biramous	Biramous	Biramous
4th leg			Uniramous	Uniramous	Uniramous	Uniramous
5th leg				Vestigial	Vestigial	Vestigial
Sternal furca					Vestigial	Present
H-suture					Vestigial?	Present
Lunulae						Present
Marginal membrane	Absent	Absent	Absent	Absent	Absent	Present
Pinnate setae on swimming legs						Present
Post-antennary process						Present

* Extension lobe of chalimus 2 completely engulfing the lobe of chalimus 1.

**Table 2:** Comparison of diagnostic features of developmental stages of female *Lepeophtheirus elegans* (based on Venmathi Maran et al. [11]).

Features	Copepodid I	Copepodid II (=Chalimus 1)	Copepodid III (=Chalimus 2)	Copepodid IV (=Preadult 1)	Copepodid V (=Preadult 2)	Adult
Body	Highly pigmented, no frontal filament	No pigmentation; short frontal filament	No pigmentation; developed frontal filament	Well-developed frontal plates	Fully developed cephalothorax	Typical caligiform cephalothorax
Cephalothorax	About 1.5 times longer than free posterior somites	About 2.5 to 3 times longer than free posterior somites	About 3.5 times longer than free posterior somites	Typical H-shaped suture	About 2 times longer than free posterior somites	As in preceding stage
Antennule (2 segments)	Proximal: 3 setae Distal: 11 setae + 2 Aesthetascs (A)	Proximal: 7 Distal: 12 + 2A	Proximal: 13; Distal: 12 + 2A	Proximal: 20; Distal: 12 + 2A	Proximal: 27 Distal: 12 + 2A	Proximal: 27 Distal: 12 + 2A
Antenna	Three-segmented; second segment with rugose process; third segment with recurved claw	Modified from that of preceding copepodid stage; third segment with curved distal claw	Middle segment with rudiment of dorsal adhesion pad	Second segment with reniform adhesion pad; terminal claw strongly curved	As in preceding stage	Terminal part forming strong, recurved claw
Maxilliped	Distal segment separated by partial suture, carrying terminal claw and trifid setal element	Distal segment of subchela bearing curved claw and short inner seta	Segments comprising subchela more completely fused than in preceding stage	As in preceding stage	As in preceding stage	Distal subchela with trace of suture separating short apical claw
Sternal furca	Absent	Absent	Present	Broad box and divergent, slightly tapering tines	As in preceding stage	As in preceding stage

**Table 3:** Overview of life cycle studies of caligid copepod species of the genera *Caligus* and *Lepeophtheirus*. Stage interpretation was confirmed by reference to Table 1 and Table 2.

Reference	Species	Stages Originally Found/Determined	Stages Interpreted and Comments
Russel [25]	*C. pageti*	N, C1, C2, Ch (“three sizes”)	N I, C I, C II, [—], [—], C V, Ad
Argilas [26]	*C. pageti*	N, C, Ch	N I, C I, [—], [—], CIV, Ad
Gurney [24]	*C. centrodonti*	N1, N2, C, Ch1, Ch2, Ch3, Ch4, Ad	N I, N II, C I, C II, [—], C IV, C V, Ad
Heegaard [29]	*C. curtus*	N1, N2, C 1, C2, pupa, Ch1, Ch2, Ch3, Ch4, Ch5, Ad	N I, N II, C I, C II, [—], C IV(?), C V(?), Ad
Kozikowska [31]	*C. lacustris*	C, Ch1, Ch2, Ch3, Ch4, Ad	[N I?], [N II?], C I, C II, [—], C IV(?), C V(?), Ad
Hwa [34]	*C. orientalis*	N1, N2, C, Ch1, Ch2, Ch3, Ch4, Ch5, Ad	N I, N II, C I, C II, C III, C IV, CV, Ad
Izawa [35]	*C. spinosus*	N1, N2, C, Ch1, Ch2, Ch3, pAd1, pAd2, Ad	N I, N II, C I, C II, C III, C IV, [--], Ad
Kabata [30]	*C. clemensi*	N1, N2, C, Ch1, Ch2, Ch3, Ch4, pAd1, Ad	N I, N II, C I, C II, C III, C IV, CV, Ad
Caillet [38]	*C. minimus*	N1, N2, C, Ch1, Ch2, Ch3, Ch4 Ch5, Ad	N I, N II, C I, [—], C III, C IV, CV, Ad
Ben Hassine [40]	*C. pageti*	N1, N2, C, Ch1, Ch2, Ch3, Ch4 pAd1, Ad	N I, N II, C I, C II, [—], C IV, CV, Ad
Hogans and Trudeau [44]	*C. elongatus*	N1, N2, C, Ch1, Ch2, Ch3, Ch4 pAd1, Ad	N I, N II, C I, C II, [—], C IV, CV, Ad
Ogawa [51]	*C. longipedis*	N1, N2, C, Ch1, Ch2, Ch3, Ch4 pAd1, Ad	N I, N II, C I, C II, C III, C IV, CV, Ad
Kim [53]	*C. punctatus*	N1, N2, C, Ch1, Ch2, Ch3, Ch4, Ad	N I, N II, C I, C II, C III, C IV, CV, Ad
Lin et al. [57]	*C. epidemicus*	N1, N2, C, Ch1, Ch2, Ch3, Ch4, Ch5, Ch6, pAd, Ad	N I, N II, C I, C II, C III, C IV, CV, Ad
Piasecki [63]	*C. elongatus*	N1, N2, C, Ch1, Ch2, Ch3, Ch4, Ad	N I, N II, C I, C II, C III, C IV, CV, Ad
Lin et al. [65]	*C.* *rotundigenitalis*	N1, N2, C, Ch1, Ch2, Ch3, Ch4, pAd, Ad	N I, N II, C I, C II, C III, C IV, CV, Ad
González and Carvajal [68]	*C. rogercresseyi*	N1, N2, C, Ch1, Ch2, Ch3, Ch4, Ad	N I, N II, C I, C II, C III, C IV, CV, Ad
Ohtsuka et al. [33]	*C. fugu*	N1, N2, C, Ch1, Ch2, Ch3, Ch4, Ad	N I, N II, C I, C II, C III, C IV, CV, Ad
Madinabeitia and Nagasawa [70]	*C. latigenitalis*	C, Ch1, Ch2, Ch3, Ch4, Ad	[N I], [N II], C I, C II, C III, C IV, CV, Ad
Khoa et al. [73]	*C. minimus*	N1, N2, C, Ch1, Ch2, Ch3, Ch4, Ad	N I, N II, C I, C II, C III, C IV, CV, Ad
Bravo et al. [42]	*C. rogercresseyi*	N1, N2, C, Ch1, Ch2, Ch3, Ch4, Ad	N I, N II, C I, C II, C III, C IV, CV, Ad
White [28]	*L. salmonis*	C, Ch1, Ch2, […], Ch(final), […], Ad	C I, C II, [—], C IV(?), C V(?), Ad
Lewis [32]	*L. dissimulatus*	N1, N2, C, Ch1, Ch2, Ch3, Ch4, Ch5, Ch6, Ad	N I, N II, C I, C II, C III, C IV, CV, Ad
Voth [36]	*L. hospitalis*	N1, N2, C, Ch1, Ch2, Ch3, Ch4, Ch5, Ch6, A	N I, N II, C I, C II, C III, C IV, CV, Ad
Boxshall [37]	*L. pectoralis*	N1, N2, C, Ch1, Ch2, Ch3, Ch4 pAd1, pAd2, Ad	N I, N II, C I, C II, C III, C IV, CV, Ad
Johnson and Albright [49]	*L. salmonis*	N1, N2, C, Ch1, Ch2, Ch3, Ch4 pAd1, pAd2, Ad	N I, N II, C I, C II, C III, C IV, CV, Ad
Schram [54]	*L. salmonis*	N1, N2, C, Ch1, Ch2, Ch3, Ch4 pAd1, pAd2, Ad	N I, N II, C I, C II, C III, C IV, CV, Ad
Venmathi Maran et al. [11]	*L. elegans*	N1, N2, C, Ch1, Ch2, pA1, pA2, Ad	N I, N II, C I, C II, C III, C IV, CV, Ad
Hamre et al. [12]	*L. salmonis*	N1, N2, C, Ch1, Ch2, pA1, pA2, Ad	N I, N II, C I, C II, C III, C IV, CV, Ad
Bravo et al. [42]	*L. mugiloidis*	N1, N2, C, Ch1, Ch2, Ch3, Ch4 pAd1, pAd2, Ad	No details available. Interpretation not possible

[—] denotes stages apparently overlooked by the cited author. [N I] and [N II] denote stages apparently known to the author but not studied. […] denotes stages that were not explicitly defined and/or named.

## Data Availability

Not applicable.
